# Visual Analysis of Odor Interaction Based on Support Vector Regression Method

**DOI:** 10.3390/s20061707

**Published:** 2020-03-19

**Authors:** Luchun Yan, Chuandong Wu, Jiemin Liu

**Affiliations:** 1 School of Materials Science and Engineering, University of Science and Technology Beijing, Beijing 100083, China; yanluchun@126.com; 2 School of Chemistry and Biological Engineering, University of Science and Technology Beijing, Beijing 100083, China; dong1035@126.com

**Keywords:** odor intensity, odor evaluation, machine learning, prediction model, antagonism effect

## Abstract

The complex odor interaction between odorants makes it difficult to predict the odor intensity of their mixtures. The analysis method is currently one of the factors limiting our understanding of the odor interaction laws. We used a support vector regression algorithm to establish odor intensity prediction models for binary esters, aldehydes, and aromatic hydrocarbon mixtures, respectively. The prediction accuracy to both training samples and test samples demonstrated the high prediction capacity of the support vector regression model. Then the optimized model was used to generate extra odor data by predicting the odor intensity of more simulated samples with various mixing ratios and concentration levels. Based on these olfactory measured and model predicted data, the odor interaction was analyzed in the form of contour maps. This intuitive method showed more details about the odor interaction pattern in the binary mixture. We found that that the antagonism effect was commonly observed in these binary mixtures and the interaction degree was more intense when the components’ mixing ratio was close. Meanwhile, the odor intensity level of the odor mixture barely influenced the interaction degree. The machine learning algorithms were considered promising tools in odor researches.

## 1. Introduction

As one of the important environmental pollution problems, odor pollution will cause people potential health hazards and uncomfortable feelings. Many countries have published related laws and regulations about the emission limits in conventional pollution sources like chemical industries, livestock processing industries, and sewage treatment systems [1]. Although, sometimes the exhaust gas has met the required emission limits but its odor is still very obvious. This phenomenon mainly comes from the superposition effect and other possible interactions caused by the mixing of multiple low-concentration odor pollutants [2]. In these cases, the olfactory evaluations by specialized human assessors are often used [3]. However, the test cost (e.g., a panel of specialized assessors, professional testing laboratory, long sampling, and testing cycles) of olfactory evaluation is much higher than regular chemical analysis [4]. Therefore, the relationship between the chemical composition and the odor intensity of odor mixtures have been widely researched in related fields [5]. The odor intensity prediction models and electronic nose are also urgently needed for both research and application purposes [6]. 

For individual odorants, the Weber–Fechner law and Power Law model have been widely used for the conversion of chemical concentration and odor intensity [7]. The linear relationship between odor activity value (ratio of chemical concentration to its odor threshold) and odor intensity is also proposed for odor intensity prediction [8]. For odor mixtures, many models like the ERM model, Vector model, U model, Additivity model, and their extended versions are reported [9,10,11]. These models provide valuable ideas and guidance for our understanding of the odor interaction. Sometimes, they also can be used in odor intensity prediction. In general, their prediction accuracy and applicable scope are often limited [12]. Even though some models have achieved accurate odor intensity prediction, the key parameters in the model must need to be experimentally measured for target substances [13]. It severely limits the convenience and practical application of these methods. Besides, these empirical models mainly focus on the mathematical associations among the target variables. The display of odor interaction law is abstract, and its feasibility to simulate and analyze influencing factors is weak [14]. Therefore, researchers are always trying to find more effective methods to investigate odor interaction. For instance, Teixeira et al. applied the perfumery ternary diagram (PTD) and perfumery quaternary diagram (PQD) methodologies to map the predicted odor intensities of fragrance mixtures [15].

In recent years, machine learning methods have developed rapidly in their algorithms and implementation techniques. Beyond traditional computer science, machine learning methods are increasingly being used for scientific researches in fields like chemistry, materials, and biology [16]. There are two important advantages of machine learning: excellent data analysis and processing capabilities to both the big data (a large amount of data and variables) and the small sample dataset (which is also a typical feature of odor data) and functional generalization capacity to new samples [17]. For example, Arabgol et al. used the support vector machine and 160 water samples data to build a nitrate concentration prediction model, and it successfully predicted the map of nitrate concentration for all four seasons [18]. Besides, machine learning also provides rich visual data analysis and utilization techniques [19]. These advantages are precisely the functions that we want in the odor interaction research. Machine learning methods have also attracted the attention of odor researchers and have been applied in various forms [20]. Szulczynski et al. proposed an electronic nose and the odor intensity was directly linked to the results of analytical air monitoring with a fuzzy logic algorithm [21]. Zhu et al. made an accurate prediction of soil organic matter contents by employing back-propagation neural network, support vector regression, and partial least squares regression methods [22]. Thus, we think that machine learning methods can also provide more useful tools in the study of odor interaction. 

In this paper, odor data of binary esters, aldehydes, and aromatic hydrocarbons mixtures were collected from our previous studies. The support vector regression algorithm was employed to establish the odor intensity prediction model which achieved the direct conversion from the mixture’s chemical composition to its odor intensity. The optimized model was used to produce more olfactory evaluation data of similar odor mixtures. Based on these odor data, we proposed a visual analysis method of odor interaction. The influences of the components’ mixing ratio and the sample concentration level on odor interaction were investigated intuitively. With the help of machine learning methods, we hope to find more effective and intuitive analysis methods of odor interaction.

## 2. Materials and Methods

### 2.1. Stimuli and Odor Data

As typical odor pollutants in odor sources like landfills and sewage treatment plants, odor data (i.e., odor threshold, Table 1; olfactory measured odor intensity and chemical concentration) of ethyl acetate (EA), butyl acetate (BA), ethyl butyrate (EB), propionaldehyde (PA), *n*-valeraldehyde (VA), *n*-heptaldehyde (HEP), benzene (B), toluene (T), ethylbenzene (E), and some of their binary mixtures were collected from our previous studies [23,24,25]. In these experiments, the odor samples were prepared through transferring a certain amount of standard gas to an odor-free plastic bag (3 L volume; Sinodour, Tianjin, China) and diluted with purified air. A sensory panel (8–14 human assessors) was employed to measure the odor threshold of each stimulus and the odor intensity of odor samples. The ASTM odor intensity referencing scale (OIRS, water solutions of 1-butanol from level 1 (aqueous solution of 12 ppm) to level 8 (1550 ppm) with a geometric progression of two at 27 ± 1 °C) was used as the standard in odor intensity evaluation [26]. More details about the testing procedure and environmental requirements were described in these references. In this study, the collected dataset contains 31 samples of binary mixture EA+BA, 21 samples of EA+EB, 22 samples of BA+EB, 24 samples of PA+VA, 24 samples of PA+HEP, 24 samples of VA+HEP, 34 samples of B+T, 31 samples of B+E, and 24 samples of T+E.

### 2.2. Support Vector Regression Methodology

Rooted in statistical learning or Vapnik-Chervonenkis (VC) theory, support vector machines (SVMs) are well positioned to generalize on yet-to-be-seen data [27]. The SVMs are very popular and effective in solving classification problems. The SVMs algorithm aims at establishing a clear gap (in the form of a line/hyperplane) that is as wide as possible to divide the sample points in a certain space. For non-linear separable data, a kernel trick is employed to map these data into high-dimensional feature spaces where the data can be successfully separated by a hyperplane. The parameters of the SVMs model will be optimized to find an optimal hyperplane that maximizes the margin of the decision boundary. The Support Vector Regression (SVR) uses the same principles as the SVMs, except that the hyperplane optimization focuses on covering as many data points as possible within a fixed-width boundary [28]. In the SVR model, the radial basis function (RBF) is mostly chosen as the kernel function, which is demonstrated quite effective in transforming non-linear data [29]. As two key parameters influencing the RBF kernel, *C* is to control the punishment degree of sample error, and *γ* is whether the accuracy is allowed to be greater than or equal to 1 for the samples of misclassification [30]. The working mechanism, mathematical formulas and optimization strategies of the SVR model have been abundantly reported in the literature [31]. Generally, the SVR model has high accuracy and excellent generalization capacity for small sample data. These advantages perfectly match the data characteristics and analysis requirements of common odor data.

In this study, the collected dataset of each binary mixture was randomly divided into two parts: the training set (70% amount) and the test set (30% amount). The training set was used in the model training and optimization steps. The chemical concentration value of each substance was used as the input variables of the SVR model, and their mixture’s olfactory measured odor intensity value was the model’s target output variable. The grid search scheme was used to search the model’s hyperparameters (the RBF kernel was used, hyperparameters C and γ were optimized), and the 10-fold cross-validation was used to evaluate the prediction ability of a model with certain hyperparameters [32,33]. Based on an optimized SVR model, the odor intensity value of a mixture can be directly predicted after inputting its corresponding chemical composition to the model. Finally, the model’s predictive capacity to new data would be verified by the test set. All of the above statistical analysis and data mining work was conducted using Python software and scikit-learn toolkit. For the main modeling and simulation content in this study, we uploaded the corresponding Python code in ipynb file format as the Supplementary Materials.

### 2.3. Experimental Procedure

This study firstly established SVR models for the odor intensity prediction of binary mixtures of esters, aldehydes and aromatic hydrocarbons. After the SVR model parameters were optimized, its predictive performance was verified by comparing the olfactory measured odor intensity with the SVR predicted odor intensity values. To evaluate the accuracy and precision of the SVR model, the coefficient of determination (*R*^2^) and the mean absolute error (*MAE*) were individually calculated for training samples and test samples. The optimized SVR models were then used to predict the odor intensity for other similar odor mixtures, and then more odor data was produced. Based on the accumulated odor data, the following strategy was used to explore the odor interaction in a more intuitive way. Here, the odor intensity value of individual substance (*OI*) was calculated on the basis of our previous obtained *OI*-ln*OAV* (natural logarithm of odor activity value) equations [24,25,26] and odor threshold values (*C_thr._*) in Table 1:

(1) $$\ln OAV=\ln\left(\frac{C}{C_{thr.}}\right)$$

(2) $$\begin{array}{cc} \mathrm{For}\;\mathrm{esters}: & \\ \; & OI=1.40\cdot \ln OAV-2.70 \end{array}$$

(3) $$\begin{array}{cc} \mathrm{For}\;\mathrm{aldehydes}: & \\ \; & OI=1.76\cdot \ln OAV-1.82 \end{array}$$

(4) $$\begin{array}{cc} \mathrm{For}\;\mathrm{aromatic}\mathrm{hydrocarbon}: & \\ \; & OI=1.07\cdot \ln OAV \end{array}$$

For binary mixtures without odor interaction, its ideal odor intensity was defined as *OI*_sum._:

(5) $$OI_{\mathrm{sum}.}=OI_{a}+OI_{b}$$

where, the value of *OI*_a_/*OI*_b_ was calculated on the basis of its chemical concentration in the mixture (Equations (1)–(4)). Both the olfactory measured odor intensity and the SVR predicted odor intensity of binary odor mixture were all marked as *OI*_mix__._, and we defined the odor interaction degree in the mixture as *OI reduction* and *OI reduction ratio*:

(6) $$OI\;reduction=OI_{\mathrm{sum}.}-OI_{\mathrm{mix}.}$$

(7) $$OI\;reduction\;ratio=\frac{OI_{\mathrm{sum}.}-OI_{\mathrm{mix}.}}{OI_{\mathrm{sum}.}}$$

Both *OI reduction* and *OI reduction ratio* were considered as indicators of interaction degree in the binary mixture. Based on these two variables, their relationships with the component’s concentration and components’ mixing ratio were carefully investigated. The contour maps and scatters plots were employed to display the odor interaction pattern of binary mixtures intuitively. 

## 3. Results and Discussion

### 3.1. Odor Intensity Predictive Performance of the SVR Model

**Figure 1:**
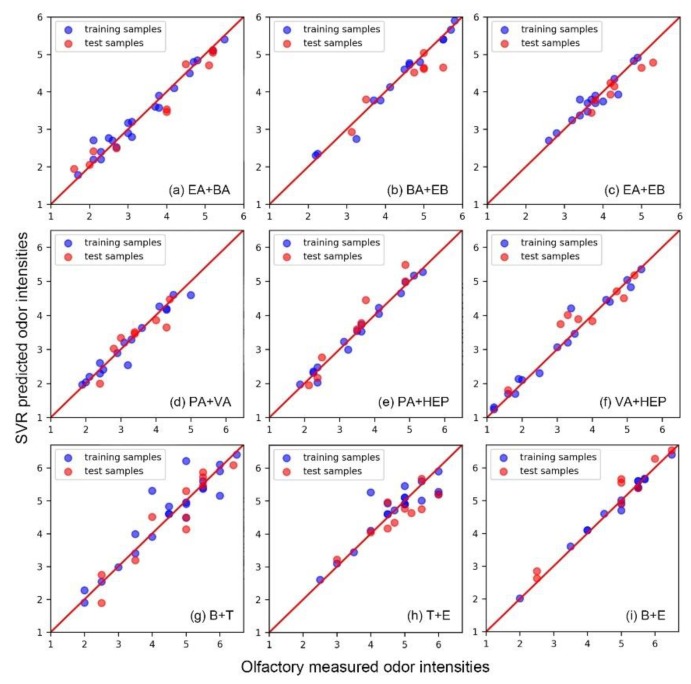
The comparison between olfactory measured odor intensity and Support Vector Regression (SVR) predicted the odor intensity of nine different binary mixtures.

As shown in Figure 1, the olfactory measured odor intensity and the SVR predicted odor intensity were compared in the form of scatter plots. When the sample point was close to the diagonal (red line), it meant that the SVR model made an accurate prediction. For most of the training samples and test samples, the SVR models successfully made perfect predictions. It demonstrated the feasibility and good fitting ability of the SVR algorithm in regular odor data analysis. Besides, the similar predictive accuracy between training samples and test samples proved that these SVR models were not overfitted. The overfitted model usually will correspond exactly to the training set, and may, therefore, fail to predict future observations reliably (like the test sample). This phenomenon is usually caused by the strong fitting ability of machine learning algorithms and its improper parameter settings, which is one of the key issues that should be avoided in the application of machine learning methods [34]. Table 2 listed the coefficient of determination (*R*^2^) and mean absolute error (*MAE*) between olfactory measured odor intensity and SVR predicted odor intensity of each odor mixture individually. From the *R*^2^ values of training samples and test samples, it also confirmed that the SVR models had good predictive accuracy and it was not over-fitted. Different from the other mixtures, the *R*^2^ values of mixture T+E was lower. It probably was caused by a relatively poor accuracy of the olfactory measured results. Because the SVR algorithm is very sensitive to the noise in the training data [35]. Therefore, the noise (arising from the error of olfactory evaluation) in the training samples can easily affect the fitting effect of the SVR model. Nevertheless, the *MAE* results still showed that the prediction error of the SVR models was very limited. In the regular olfactory evaluation tests, the 0.4 OIRS level of error was usually observed and widely accepted [25]. Thus, the optimized SVR models were considered to be useful and accurate in the odor intensity prediction of these binary odor mixtures. 

The odor intensity prediction models were considered promising techniques in the field of odor evaluation. First, the prediction models could directly perform the odor intensity evaluation (basing on the composition and concentration information measured by analytical equipment) instead of human assessors. The influences of assessor quantity, age, gender, and testing environments could be avoided [36]. On the other hand, it has been reported that the e-nose can directly perform odor intensity evaluation [21,37]. However, it directly correlates the sensor signal and odor intensity, and does not fully consider the gas mixture’s composition. Therefore, the device is more focused to specific target gases. In contrast, e-noses and online monitoring devices capable of gas identification and concentration detection are more common and more mature [38,39]. If combining the odor intensity prediction model with these e-noses and online monitoring devices, it will significantly improve their olfactory assessment capacity and extend the applicable scope.

### 3.2. SVR-Assisted Visual Analysis of Odor Interaction

**Figure 2:**
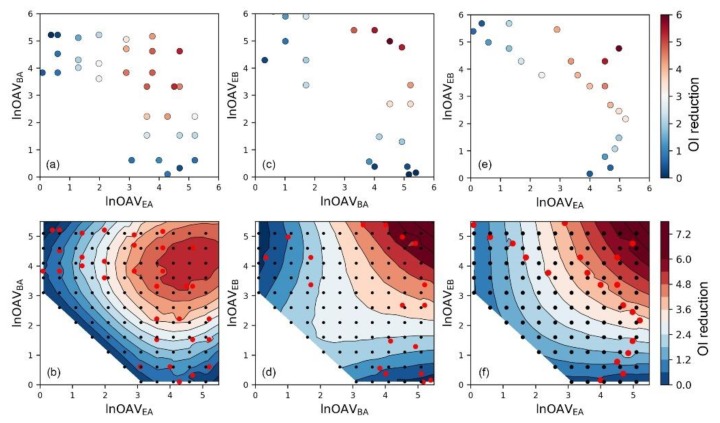
The scatter plot of the relationship between olfactory measured odor intensity reduction degree (*OI reduction*) and components’ ln*OAV* (natural logarithm of odor activity value) values, and corresponding contour map after adding SVR model predicted data (in the contour map, red dots: olfactory measured data; black dots: SVR predicted data) for binary mixture (**a**,**b**) EA+BA, (**c**,**d**) BA+EB, and (**e**,**f**) EA+EB.

In comparison with traditional odor intensity prediction models, some machine learning methods like SVR have a significant advantage and also one of its disadvantages. The mechanism like a black box severely limited its function in explaining related mechanisms and laws. Although many studies have established empirical models to explain the odor interaction phenomenon and made conclusions, we still hope to develop more analytical methods through the reasonable use of machine learning methods. Since it has been proved that there is a simple linear relationship between *OI* and ln*OAV* of an individual substance, we think that using the ln*OAV* value to represent the content of a component is also helpful in odor interaction studies [23,24]. As illustrated in Figure 2a, c, and d, scatter plots of the relationship between each component’s content (in the form of ln*OAV*) and the odor interaction degree (in the form of *OI reduction* values; i.e., the color of each dot) was plotted. Based on the definition of *OI reduction* in Equation (6), the larger *OI reduction* value meant the stronger degree of antagonism effect [40]. It could be seen that when the content of both components was high, the antagonism effect would be more intense. Because the amount of actual olfactory measured odor data was limited, this scatters plot only provided little information and the results were not intuitive enough. 

In order to obtain more odor evaluation results, we used the SVR model instead of olfactory measurement which saved much time and economic costs. Since the core idea of machine learning was to find out the mathematical relationship between the chemical composition and the corresponding mixture’s odor intensity, a certain amount of training samples usually could guarantee the modeling effect. After that, the optimized model would also be valid for other similar samples. This strategy and function have been fully demonstrated and widely applied in many research [41]. As shown in the above *R*^2^ and *MAE* analysis results of the training and test samples, the SVR model had obtained the correct mapping relationship. In this case, the optimized model also will be valid for other samples of the corresponding mixture with different chemical concentrations. As shown by the black dots plotted in Figure 2b,d,f, we predicted the odor intensity of many binary mixtures with different chemical contents. Results from actual olfactory measurements in our previous studies were plotted with red dots. In order to distinguish the data source here, the color of the dots no longer indicates the *OI reduction* value like Figure 2a,c,e. Based on these olfactory measured and SVR predicted results, the contour maps about the *OI reduction* degree and mixtures’ composition were plotted. Through these diagrams, the interaction of odor substances became more intuitive. It could be concluded that the degree of antagonism effect was usually weak if the ln*OAV* value of any component is low. When the content of one component was constant, the antagonism degree would increase as the content of the other component enhancing. Besides, the antagonism degree would become more intense when the ln*OAV* values of the two components were approaching close. 

**Figure 3:**
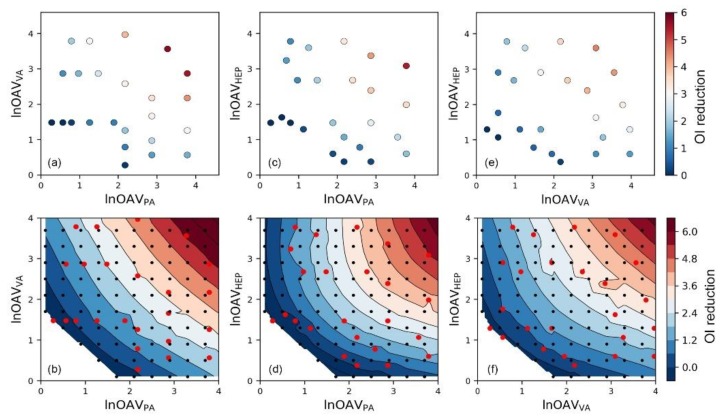
The scatter plot of the relationship between olfactory measured odor intensity reduction degree (*OI reduction*) and components’ ln*OAV* values, and corresponding contour map after adding SVR model predicted data (in the contour map, red dots: olfactory measured data; black dots: SVR predicted data) for binary mixture (**a**,**b**) PA+VA, (**c**,**d**) PA+HEP, and (**e**,**f**) VA+HEP.

In the same way, the odor interaction of binary aldehydes mixtures was also analyzed (Figure 3). The interaction pattern of binary aldehydes mixtures was almost the same with esters mixtures. However, we could still see that there were some differences in the details of their contour maps. For instance, the antagonism degree of mixture BA+EB (Figure 2d) was weaker when the ln*OAV*_BA_ and ln*OAV*_EB_ values were close to 2–3. So did the mixture EA+BA when the ln*OAV*_EA_ and ln*OAV*_BA_ values were close to 4.5–5.5 (Figure 2b). A very obvious difference was that there were almost no olfactory measured sample data in the areas mentioned above. Because there were not enough samples to reflect the real odor interaction in these areas, the performance of the SVR model to the corresponding area was easily affected by other samples. In machine learning, this phenomenon is generally observed because of insufficient sample amount and lacking data representativeness [42]. The samples in the aldehyde mixtures were more evenly dispersed, so the odor interaction in each area was fully reflected. Therefore, reasonable sampling also should be concerned when using machine learning methods in odor researches. 

**Figure 4:**
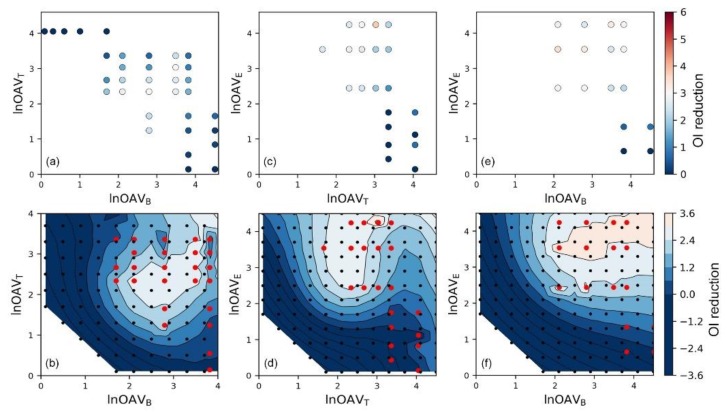
The scatter plot of the relationship between olfactory measured odor intensity reduction degree (*OI reduction*) and components’ ln*OAV* values, and corresponding contour map after adding SVR model predicted data (in the contour map, red dots: olfactory measured data; black dots: SVR predicted data) for binary mixture (**a**,**b**) B+T, (**c**,**d**) T+E, and (**e**,**f**) B+E.

The contour maps of binary aromatic hydrocarbon mixtures were plotted in Figure 4. Unlike binary mixtures of esters and aldehydes, the overall antagonism degree of binary aromatic hydrocarbon mixtures was at a relatively low level. When the ln*OAV* value of both components was higher than 2.5, a similar antagonism degree-mixing ratio relationship like esters and aldehydes was observed (Figure 4b,d,f). When their ln*OAV* values were smaller than this critical value, we observed a synergism effect (i.e., the negative *OI reduction* value which meant that the *OI*_mix._ was higher than the *OI*_sum._). Because there was no actual olfactory measured sample data in this area, the reliability of this phenomenon should be verified by further olfactory evaluation tests. In all these contour maps, the odor interaction of odor samples with too small ln*OAV* values (i.e., the lower left blank corner of the contour plot) was not considered. Because odor samples belonging to this area usually had the odor intensity value lower than 2.0 of the OIRS (it could be demonstrated from Figure 1). For those odor samples, the error of olfactory evaluation was usually higher [25]. Meanwhile, it also was more meaningful to analyze the odor interaction of odor mixtures with distinct olfactory stimulation.

### 3.3. Similarity of Binary Odor Interaction Pattern

**Figure 5:**
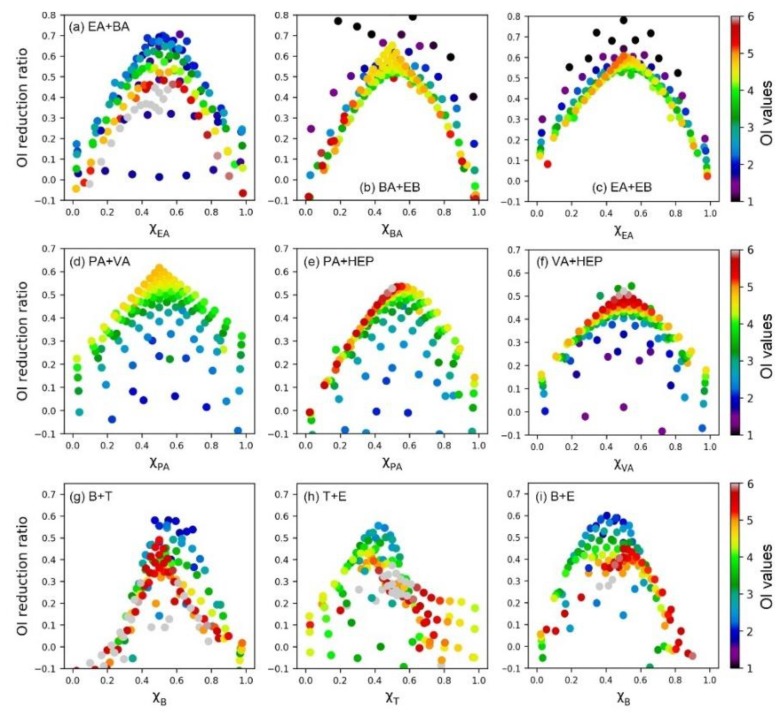
The scatter plot of *OI reduction ratio* vs. components’ mixing ratio for binary odor mixtures. Colors represent the odor intensity of each sample.

In order to further verify the above-observed odor interaction pattern, we also analyzed the influence of the sample’s odor intensity level. As depicted in Figure 5, all the olfactory measured data and SVR predicted data were employed and colors represented the odor intensity of each sample. We defined the *OI reduction ratio* (Equation (7)) and mixing ratio of the binary mixture (i.e., *x*_a_ = ln*OAV*_a_/(ln*OAV*_a_ + ln*OAV*_b_)). Firstly, we observed the same conclusion as the above contour maps. When the ln*OAV* mixing ratio of the two components was close, the antagonism degree was the most obvious (i.e., higher *OI reduction ratio*). Secondly, most of the odor samples followed the same odor interaction pattern regardless of its specific odor intensity level. No significant correlation was observed between the *OI reduction ratio* and the sample’s odor intensity value. It was consistent with the phenomenon observed in our previous PDE (partial differential equation) model researches [25]. Compared with the influence of the sample’s odor intensity value, the odor interaction degree was mainly affected by the components’ mixing ratio. 

In this study, we mainly employed the SVR algorithm as a useful tool for data analysis. Based on its strong regression ability, more reliable data was collected and it helped to explore the odor interaction more intuitively. Although the odor interaction has been widely studied by many empirical models who have made very accurate explanations [5,6,11,43], the machine learning method still has distinct advantages like visual analysis and low time/economic cost. As we found in this study, enough olfactory measured data was an essential guarantee to the accuracy of machine learning models. When applying the machine learning methods, we also should pay attention to the sample representativeness and the objective analysis of simulation results. On the other hand, the observed odor interaction pattern was only verified by several substances from the esters, aldehydes, and aromatic hydrocarbon groups. In order to further prove the reliability and applicable scope of currently observed odor interaction pattern, it is necessary to test more odor substances. 

## 4. Conclusions

In this study, a support vector regression algorithm was employed to train an odor intensity prediction model of binary esters, aldehydes, and aromatic hydrocarbon mixtures individually. The chemical concentrations of each component were directly transformed into the odor intensity of the mixture, and it successfully avoided the interference from odor threshold measurement and individual components’ odor intensities evaluation which was usually performed by human assessors. The optimized model showed high accuracy for both the training samples and test samples. It was also considered adequate for other odor mixtures with different mixing ratios and concentration levels. Based on the support vector regression model, more odor data were collected and these data supported the visual analysis of odor interaction. Compared with traditional empirical models, the visual analysis method was more intuitive and provided more information. A similar odor interaction pattern was observed among these binary odor mixtures. Meanwhile, the importance of original olfactory measured data and data representativeness was also proved in the results. As a fast-growing technology, we have demonstrated its potential in the odor interaction analysis. 

## Figures and Tables

**Table 1:** List of odorants and their odor thresholds.

Order	Odorant (Abbreviation)	CAS#	Chemical Structure	Odor Threshold/mg/m^3^
1	ethyl acetate (EA)	141-78-6	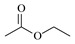	0.276 ^I^
2	butyl acetate (BA)	123-86-4	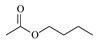	0.085 ^I^
3	ethyl butyrate (EB)	105-54-4	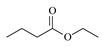	0.053 ^I^
4	propionaldehyde (PA)	123-38-6	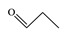	40.6 E-3 ^II^
5	*n*-valeraldehyde (VA)	110-62-3	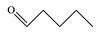	20.5 E-3 ^II^
6	*n*-heptaldehyde (HEP)	117-71-7	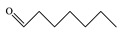	26.0 E-3 ^II^
7	benzene (B)	71-43-2	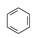	2.53 ^III^
8	toluene (T)	108-88-3	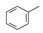	1.43 ^III^
9	Ethylbenzene (E)	100-41-4	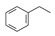	0.45 ^III^

^I^ Odor detection thresholds in reference [24]; ^II^ Odor detection thresholds in reference [26]; ^III^ Odor detection thresholds in reference [25].

**Table 2:** The prediction accuracy of SVR models. The coefficient of determination (*R*^2^) and mean absolute error (*MAE*) were individually calculated for each kind of binary mixture.

Mixture	*R* ^2^		*MAE*	
	Training Set	Test Set	Training Set	Test Set
EA+BA	0.97	0.95	0.15	0.26
BA+EB	0.96	0.85	0.15	0.33
EA+EB	0.87	0.87	0.17	0.25
PA+VA	0.95	0.78	0.14	0.25
PA+HEP	0.96	0.94	0.17	0.23
VA+HEP	0.97	0.87	0.15	0.31
B+T	0.87	0.81	0.33	0.43
T+E	0.78	0.68	0.31	0.40
B+E	0.98	0.94	0.09	0.27

## Supplementary Materials

The following are available online at https://www.mdpi.com/1424-8220/20/6/1707/s1, Code S1: Code of SVR model for odor interaction simulation.ipynb.
